# Optimising recruitment to the HAND-1 RCT feasibility study: integration of the QuinteT Recruitment Intervention (QRI)

**DOI:** 10.1186/s40814-020-00710-1

**Published:** 2020-11-09

**Authors:** Samantha Husbands, Daisy Elliott, Tim R. C. Davis, Jane M. Blazeby, Eleanor F. Harrison, Alan A. Montgomery, Kirsty Sprange, Lelia Duley, Alexia Karantana, William Hollingworth, Nicola Mills

**Affiliations:** 1 Bristol Medical School, University of Bristol, 1-5 Whiteladies Road, Bristol, BS8 1NU UK; 2 Bristol Medical School, University of Bristol, Canynge Hall, 39 Whatley Road, Bristol, BS8 2PS UK; 3 Nottingham University Hospitals NHS Trust, Queen’s Medical Centre, Derby Road, Nottingham, NG7 2UH UK; 4 Nottingham Clinical Trials Unit, University of Nottingham, University Park, Nottingham, NG7 2RD UK; 5 Department of Academic Orthopaedics, Trauma and Sports Medicine, School of Medicine, University of Nottingham, Nottingham, NG7 2UH UK

**Keywords:** Feasibility study, Randomised controlled trial, Surgical randomised controlled trial, Recruitment, Recruitment obstacles, Recruitment intervention, Informed consent, Qualitative, Equipoise

## Abstract

**Background:**

Recruitment to randomised controlled trials (RCTs) can be challenging, with most trials not reaching recruitment targets. Randomised feasibility studies can be set up prior to a main trial to identify and overcome recruitment obstacles. This paper reports on an intervention—the QuinteT Recruitment Intervention (QRI)—to optimise recruitment within a randomised feasibility study of surgical treatments for patients with Dupuytren’s contracture (the HAND-1 study).

**Methods:**

The QRI was introduced in 2-phases: phase 1 sought to understand the recruitment challenges by interviewing trial staff, scrutinising screening logs and analysing audio-recorded patient consultations; in phase 2 a tailored plan of action consisting of recruiter feedback and training was delivered to address the identified challenges.

**Results:**

Two key recruitment obstacles emerged: (1) issues with the recruitment pathway, in particular methods to identify potentially eligible patients and (2) equipoise of recruiters and patients. These were addressed by liaising with centres to share good practice and refine their pathway and by providing bespoke feedback and training on consent discussions to individual recruiters and centres whilst recruitment was ongoing. The HAND-1 study subsequently achieved its recruitment target.

**Conclusions:**

Transferable lessons learnt from the QRI in the feasibility study will be implemented in the definitive RCT, enabling a “head start” in the tackling of wider issues around screening methods and consent discussions in the set up/early recruitment study phases, with ongoing QRI addressing specific issues with new centres and recruiters. Findings from this study are likely to be relevant to other surgical and similar trials that are anticipated to encounter issues around patient and recruiter equipoise of treatments and variation in recruitment pathways across centres. The study also highlights the value of feasibility studies in fine-tuning design and conduct issues for definitive RCTs. Embedding a QRI in an RCT, at feasibility or main stage, offers an opportunity for a detailed and nuanced understanding of key recruitment challenges and the chance to address them in “real-time” as recruitment proceeds.

## Key messages regarding feasibility

- What uncertainties about feasibility existed prior to this study?

Recruitment to a randomised trial to assess the effectiveness of treatment for Dupuytren’s contracture was anticipated to be challenging due to differences between procedures in the speed of recovery and risk of disease recurrence, and the likelihood of recruiting clinicians and patients having preferences towards a particular procedure.

- What are the key feasibility findings from this study?

Embedding a QuinteT Recruitment Intervention (QRI) within the HAND-1 study exposed key recruitment obstacles with regards to methods for identifying potentially eligible trial participants and individual recruiter and patient equipoise. Strategies, including sharing of findings giving examples of good practice and training on consent discussion, were implemented at individual, centre and whole study level to address the identified challenges and optimise recruitment.

- What are the implications of the feasibility findings on the design of the main study?

These findings have implications for the design and conduct of the main study including the need for early discussions with centres regarding effective (and less effective) screening methods, and upfront and continued training on consent discussions, in particular conveying equipoise and engaging with patients’ treatment preferences.

## Background

Randomised controlled trials (RCTs) are widely acknowledged as the gold standard study design for establishing the effectiveness of interventions [1]. However, recruiting to RCTs is challenging, and only half reach recruitment targets [2], with studies facing early closure or an extended recruitment period [3]. Randomised feasibility studies can help to identify problems with recruitment in advance of a definitive RCT, including barriers to participation and the identification of clinician training needs [4]. This paper reports on an intervention embedded into a feasibility hand surgery RCT that aimed to identify and overcome barriers to recruitment and informed consent prior to a main trial.

The HAND-1 study was a two-arm randomised study developed to assess the feasibility and to inform the design of a large multicentre RCT to compare the clinical and cost-effectiveness of treatments for Dupuytren’s contracture. Dupuytren’s contracture is a thickening of the connective tissue in the palm of the hand that eventually results in one or more affected fingers being permanently bent in towards the palm—adversely affecting patients’ hand function and ability to carry out everyday tasks [5]. Currently, there is a lack of high-quality evidence comparing two surgical treatments for primary Dupuytren’s contracture: limited fasciectomy (LF) and needle fasciotomy (NF) [6]. Both NF and LF are currently performed within the National Health Service (NHS) in England. LF is most commonly undertaken, but NF is less costly for health services, at least in the short term. Dupuytren’s contracture can come back after both NF and LF, such that further surgery may be needed, though recurrence is more common after NF [7].

Recruitment to the HAND-1 study was anticipated to be challenging due to (a) differences between the procedures in the speed of recovery and risks of recurrence and need for revision surgery [7, 8] and (b) the likelihood of recruiting clinicians and patients not being in equipoise and having preferences towards one or the other procedure. Clinical equipoise is the state of uncertainty which arises when no one procedure is considered to be more or less beneficial for treating a patient group than its comparators [9]. Conveying equipoise to patients during trial recruitment can be challenging and requires recruitment staff to present treatments in a balanced way and avoid unwittingly conveying any personal beliefs to patients about the superiority of any procedure, given the lack of robust evidence [10]. Ensuring that patients are well-informed about the treatment options is equally crucial and a basic requirement of good clinical practice. To enable this, recruiters need to be comfortable exploring the basis of patients’ treatment preferences to expose and address any concerns and misconceptions and ensure that trial participation decisions are based on full and accurate information [11].

Given the anticipated difficulties, a QuinteT Recruitment Intervention (QRI) was embedded into HAND-1 to optimise recruitment and informed consent. The QRI aims to rapidly identify recruitment difficulties and offers tailored solutions whilst recruitment continues [12]. Having evolved over two decades, the intervention has been implemented in over 30 RCTs, leading to insights about recruitment issues across a variety of trial contexts and the development of evidence-based strategies to address them [13–16].

This paper reports on the identification of barriers to recruitment and informed consent through the QRI, and the strategies designed and delivered to overcome them, enabling the study to recruit to target. The main results of HAND-1 feasibility study are reported separately [17].

## Methods

### The HAND-1 feasibility RCT

Patients who met the inclusion criteria [6] were recruited from three secondary care centres in England. Recruitment was undertaken by hand surgeons (lead recruiters) and research nurses (or supporting recruitment staff) between November 2015 and September 2016. Lower and upper recruitment targets of 50–85 patients were set before the study commenced. Patients who agreed to participate were randomised on a 1:1 ratio to receive either NF or LF. A range of patient reported, and clinical outcome measures were collected to test their appropriateness for the main trial [6].

### The embedded QuinteT Recruitment Intervention

The QRI has two phases. Phase 1 sought to identify and understand recruitment challenges using multiple methods, including qualitative interviews with trial staff, scrutiny of screening logs and analysis of audio-recorded consultations in which the trial was discussed and patients invited to participate. Phase 2 involved designing and implementing a tailored plan of action to address the challenges identified in phase 1. This entailed site and individual feedback, training and support. Recruitment rates were plotted over time to examine trends in recruitment and their association with the timings of QRI interventions. Data collection for phase 1 of the QRI took place soon after recruitment commenced (November 2015) until May 2016, to allow time for centres to receive training and feedback and for this to potentially impact recruitment.

### Phase 1

#### Interviews with HAND-1 trial staff

Semi-structured interviews with key trial management group (TMG) members and HAND-1 recruitment staff were undertaken by SH. Staff were invited for telephone interview via e-mail; those who did not respond were sent a reminder one week later. A flexible interview topic guide (available as supplementary material) was developed based on those used in previous QRIs [12] with questions focusing on perceived issues with recruitment and how trial processes and consultations were being conducted.

Interviews were transcribed verbatim and checked against audio-recordings for accuracy. Interview transcripts were imported into NVivo10 and coded line-by-line for emerging themes. Data were assigned representative labels and analysed using techniques of constant comparison, which requires new data to be continually compared with existing data to enhance understanding and explore relationships between themes [18]. A coding structure was developed and refined to take account of new and evolving themes as analysis progressed. Descriptive accounts were generated to compare interview responses under each theme. Analysis was primarily undertaken by SH, with 10% of transcripts double-coded by DE to enhance reliability [19].

#### Analysis of screening log data

All centres were encouraged to keep detailed screening logs of their recruitment activities, including numbers of patients screened, eligible, approached and recruited (SEAR framework) [20], and reasons for non-participation. Centres received training on completing the logs and were sent reminders every two weeks to complete them in full and upload them to the trial database. Data were collated by the Nottingham Clinical Trials Unit (NCTU) and screening logs were analysed using simple counts to display numbers and proportions of patients at each stage of the eligibility and recruitment process.

#### Recorded consultations offering participation

Patients were posted information about audio-recording their discussion with the surgeon before their NHS clinic appointment where recruitment occurred. At clinic, recruitment staff were encouraged to routinely approach all patients for permission to audio-record. Patients who agreed provided written informed consent. Recordings were captured on encrypted audio-recorders, transcribed verbatim and analysed using methods of constant comparison (as described above). Analysis focused on interaction between recruiters and patients to compare recruiter explanations of the study and patient responses. Techniques inspired by conversation analysis [21] were used to study in-depth aspects of communication that appeared to hinder recruitment. A sub-sample of appointment transcripts was double-coded by DE and NM.

#### Recruiter training in phase 1

Training recruiters is typically undertaken in phase 2 of the QRI, but opportunities arose to offer early training based on findings from previous QRIs [14]. In the second month of recruitment (13 January 2016), a training session was embedded within a collaborator’s meeting and a general recruitment tips document was circulated to all staff. The meeting was attended by surgeon C and research nurses and other supporting staff from all centres.

### Phase 2

In phase 2, findings from phase 1 were presented in a report to the chief investigator (CI) and TMG (28 February 2016). This detailed recruitment obstacles within and across centres, and presented anonymised trial staff interview, recruitment consultation audio-recordings and screening log data as evidence. Findings were discussed, and a tailored plan of action developed for each centre, focusing on the delivery of targeted recruitment feedback by the QRI team.

## Results

A total of 267 patients were assessed for eligibility between November 2015 and September 2016, of which 153 (57%) were eligible. Seventy-one (46%) of these were randomised to receive NF or LF, which was within the pre-set recruitment target range of 50–85 participants demonstrating that a main trial would be feasible.

### Interviews with HAND-1 trial staff

Eight HAND-1 staff were approached for interview, of which seven agreed. Interviews took place between December 2015 and February 2016 with one TMG member, and a recruiting surgeon (*n* = 3) and research nurse (*n* = 3) from each centre (Table 1). The lead recruiter in centre 3 was not available for interview, but another recruiter in the centre was interviewed (surgeon A). Interviews lasted 30 min on average (range 15–50 min).

**Table 1:** HAND-1 interview participants

Participant ID	Centre	Role in HAND-1 study
Surgeon A	Centre 3	Recruiter
Surgeon B	Centre 2	Lead recruiter at centre 2
Surgeon C	Centre 1	Lead recruiter at centre 1/TMG member
Research nurse A	Centre 3	Research nurse
Research nurse B	Centre 2	Research nurse
Research nurse C	Centre 1	Research nurse/assistant
TMG member A	N/A	TMG member

### Recorded consultations offering participation

Of 267 patients assessed for eligibility for the trial across the 3 centres, 115 were approached for audio-recording between December 2015 and September 2016, and 86 (74%) agreed. For this study, analysis focused on the practices of two of the three lead recruiting surgeons (surgeon B and C) from two of the centres, as they were the only recruiters with audio-recordings pre- and post-feedback. However, insights through interviews with a recruiting surgeon from the third centre were included in the phase 1 analysis. Seventy-two patients were approached for audio-recording by surgeon B and C, and 65 agreed (90%). Of these 65 recordings, 37 were made in phase 1 and 28 in phase 2, after all training had been delivered.

### Phase 1 results: key recruitment obstacles

#### Organisational issues: variation of patient pathways

**Fig. 1:**
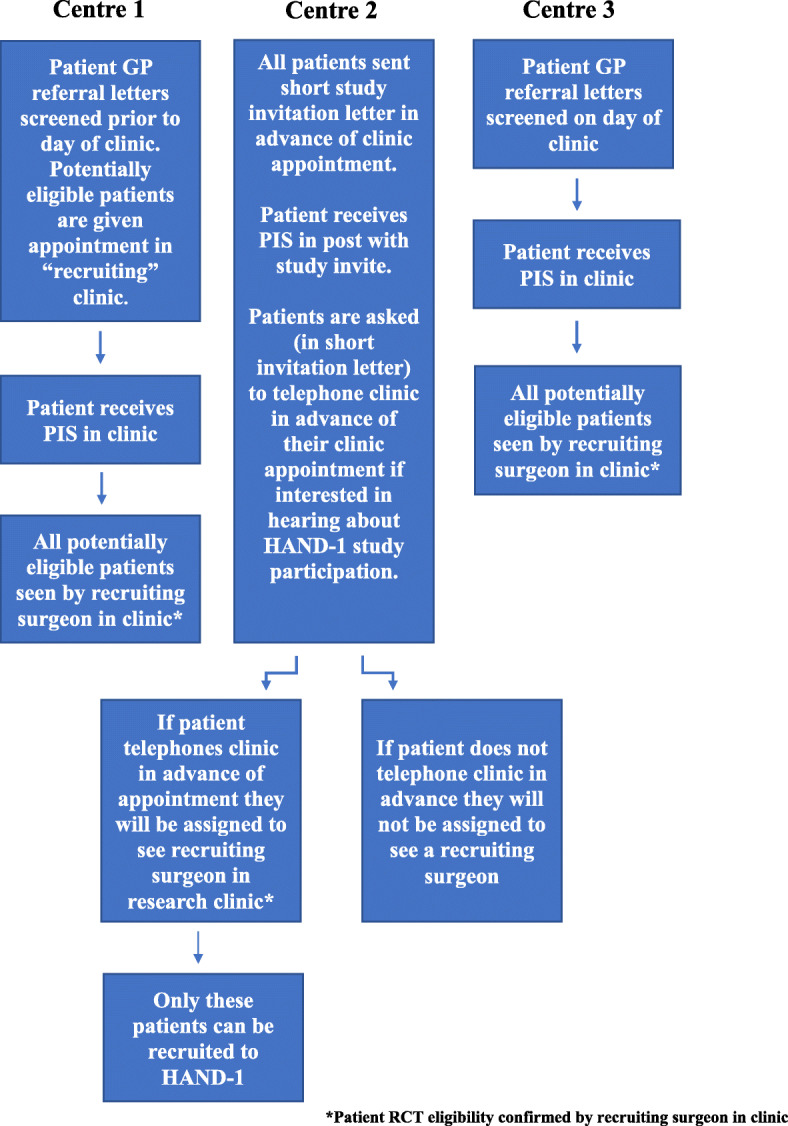
Patient recruitment pathways for HAND-1 across recruiting centres. *Patient RCT eligibility confirmed by recruiting surgeon in clinic

Recruitment figures from screening logs in the first three months of recruitment indicated differences in recruitment activity across centres. Centre 2 had screened and recruited fewer patients than centre 1 (7 screened compared to 19 and 2 recruited compared to 5), even though centre 2 had an expected higher volume of eligible patients. Descriptions of patient recruitment pathways during staff interviews highlighted differences in how patients were screened (Fig. 1), which was likely to have explained the observed differences in numbers of potentially eligible participants identified. Centre 1 screened GP referral letters to ensure all patients were guided to a “recruitment clinic” in advance, and centre 3 screened patient notes on the day of clinic. Eligible patients in both centres were given a study patient information sheet (PIS) to look at in clinic, and the opportunity to discuss the study with members of the recruitment team. At centre 2, however, centre staff opted to send a study invitation letter and leaflet by post, asking patients to call in advance of their appointment to “opt-in” to a research clinic to discuss participation in HAND-1. Opportunities for these patients to discuss the study with staff before deciding whether or not to opt-in to the research clinic were therefore limited.

#### Presenting the RCT to patients: patient preferences and surgeon equipoise

Recruitment figures around the time of staff interviews (February 2016) demonstrated that 81% of eligible patients who declined participation were doing so because they favoured either NF or LF. These figures aligned with opinions of recruitment staff, particularly recruiting surgeons, who suggested that patients would have strong views on which treatment, or aspects of treatment, would be most appropriate (Table 2). If patients voiced a preferred treatment, they suggested they would respect this and offer them their treatment of choice (Table 3). This played out in practice, with surgeons B and C tending to accept expressed treatment preferences early on in discussions, often without exploring what patients understood by the treatments and therefore how informed they were about them (Table 4). In example consultation 2 (Table 4), surgeon B missed opportunities to address a patient’s concern about scarring with LF, without also balancing this with information about the decreased likelihood of Dupuytren’s contracture reoccurring after LF compared to NF. Both patients in these examples opted to receive their initial treatment preference.

**Table 2:** Extracts from interviews: recruiters’ perception of patient equipoise

**Example 1:** **Surgeon C (centre 1, interview):** “[Patients] will have strong preferences for one or the other operation…one has a quicker recovery but is more likely not to straighten the finger fully and have to be repeated, some people will say, ‘…As long as it can be repeated, I’m happy.’ Others say, ‘…I want a once-and-for-all, if possible, treatment,’ and go for the bigger one.” **Example 2:** **Surgeon B (centre 2, interview):** “I get a lot of musicians…rock climbers…people like electricians, self-employed who are having trouble with their job, but can’t take six weeks off work with LF…So it may be that actually, equipoise is not possible because patient choice will determine LF so critically.”

**Table 3:** Extracts from interviews: recruiters’ intended response to voiced preferences

**Example 1:** **Surgeon A (centre 3, interview):** “So, obviously if they have already decided then fine, they’ll [get it]. We respect their decision…” **Example 2:** **Interviewer:** “What do you plan to do if a patient does decline [because of a preference]?” **Surgeon B (centre 2, interview):** “Offer them the treatment of their choice.” **Example 3:** **Interviewer:** “Why do you think patients might decline to take part in the trial?” **Surgeon C (centre 1, interview):** “Because of a preference…There are many reasons [why], and I think you’ve just got to accept some people will have views as to what suits them better” **Interviewer:** “What will you do if a patient does decide to decline to take part?” **Surgeon C (centre 1, interview):** “That’s fine. They will be asked which treatment they want to have…Then on the NHS they’ll have the treatment they wish…”

**Table 4:** Excerpts from recruitment consultations: recruiter responses to patient treatment preferences

**Example consultation 1 (surgeon C, centre 1):** **Patient 16:** “I think I’d like to have, would prefer to have, limited fasciectomy.” **Surgeon C:** “No, that’s absolutely fine” **Example consultation 2 (surgeon B, centre 2):** **Patient 6:** [referring to LF surgery] “When I look at all this scarring and everything, I think it looks really ugly…. And the fact is that it [Dupuytren’s contracture] will come back anyway.” **Surgeon B:** “Yes.” **Patient 6:** “I think probably I would prefer the needle….” **Surgeon B:** “Okay”	

Recruiters were also observed presenting imbalanced information on treatment options, possibly unwittingly, in the language they used, for example, in describing LF as “a major event” but NF as “something simpler” (surgeon C) or using less positive language to describe one operation “there’s LF…some people would call it bog-standard…. I don’t know it’s the best” (surgeon B). In the case of surgeon B, this imbalanced presentation appeared to be influenced by a personal preference for NF, with instances of patients being guided, inadvertently or otherwise, towards choosing NF. In these cases, the patient declined trial participation requesting NF instead (Table 5).

**Table 5:** Excerpt from recruitment consultation regarding surgeon equipoise

**Patient 17:** “When I came in today, my thought was, the recovery time was a minus [of LF]. Scars or…lumps, doesn’t bother me in the slightest. So, I suppose I had in my mind the needle option. If you were saying to me a better recovery will be surgery, I’m not anti-surgery…. I suppose I want it done as quickly as possible. I would certainly be guided by yourself.” **Surgeon B:** “Okay. This [centre] has a reputation for doing NF…and people come to us for that reason…. they say, ‘Getting back to work rapidly is my highest priority. I’m willing to put up with it coming back’.” **Patient 17’s wife:** “Ah, that was a question I was going to ask you. If [Patient 17] had it done by needle now and it did not, in time he could then have it cut and done…. Because that would give you options at work now, wouldn’t it?” **Surgeon B:** “Yes.” **Patient 17:** “Mmm-hmm” **[Patient 17 opts for Needle Fasciotomy]**	

### Phase 2: strategies implemented to address identified recruitment difficulties

The plan of action consisted of written reports to the TMG, written individual feedback to recruiters on recruitment consultations and face-to-face, centre-specific training sessions focusing on key recruitment obstacles identified in phase 1. These were delivered between February and early August 2016 (see Table 6).

**Table 6:** HAND-1 QRI interventions

Date	QRI intervention	Received by/centre	Agreed action as result of feedback
13 January 2016, phase 1	Recruiter training presentation given at collaborators’ meeting	Surgeon C, centre 1	N/A
13 January 2016, phase 1	HAND-1 recruiter tips document circulated to centres	All	N/A
28 February 2016, phase 1	Full QRI report sent to CI and TMG members (including suggested actions)	Surgeon C, centre 1 (as a member of the TMG)	N/A
10 May 2016, phase 2	Site-specific feedback visit/training to centre 1	Surgeon C, research nurse C and other staff involved with recruitment in centre 1	Carefully consider recruitment practices—specifically to explore and address treatment preferences rather than accept them at face value.
11 May 2016, phase 2	Site-specific feedback visit/training to centre 2	Surgeon B, research nurse B and other staff involved with recruitment in centre 2	Streamline patient recruitment pathway. Carefully consider recruitment practices—specifically to provide more balanced overview of procedures and explore patient preferences rather than accept them at face value.
18 May 2016, phase 2	Individual and tailored written feedback sent to recruiting surgeons	Surgeon B, surgeon C and 1 additional recruiter (surgeon E) in centre 1	N/A
22 June 2016, phase 2	Tailored tips document (based on issues identified from screening log data e.g. addressing patient treatment preferences)	All recruitment staff in centre 3	N/A
1 August 2016, phase 2	Individual and tailored written feedback	Surgeon D, centre 2	N/A

The written report to the TMG (February 2016) detailed obstacles to recruitment across the centres, based on analysis of screening logs, staff interviews (*n* = 8) and audio-recordings available at the time (*n* = 11). The report was accompanied by a list of potential actions to address issues highlighted.

Face-to-face visits to centres 1 and 2 took place in May 2016. Feedback sessions used anonymised data from HAND-1 consultations to demonstrate how different approaches to presenting study information appeared to impact patients’ decisions, for example, how presenting information in favour of one treatment over another would (often unwittingly) sway patients towards the weighted treatment. Guidance was offered on how lead recruiters could optimally manage aspects of recruitment discussions, such as techniques to explore the rationale for treatment preferences, to allow patient concerns or misconceptions to be unearthed and explored. After feedback sessions, an e-mail was sent to centres with a list of agreed actions, which included adjustments to the recruitment pathway in centre 2 and changes to aspects of recruiters’ approaches to explaining the study in centres 1 and 2 (Table 6). Changes to centre 2’s recruitment pathway involved the appointment of staff to screen clinic letters and an additional recruiting surgeon (surgeon D), allowing all patients to be screened and approached for HAND-1 participation during their clinic visit. Surgeon B and C also received confidential and tailored written feedback on their recruitment consultations, for example, highlighting how their choice of words and proportion of time spent describing treatments could unwittingly steer patients towards a particular procedure, with tips and guidance on how to overcome to present better balanced information.

Centre-specific or individual recruiter feedback could not be delivered to centre 3. Instead, a tailored written tips document on addressing patient preferences was circulated, as this centre’s screening logs demonstrated patients were declining study participation because of a preference for one of LF or NF.

### Observed changes to recruitment and informed consent

**Fig. 2:**
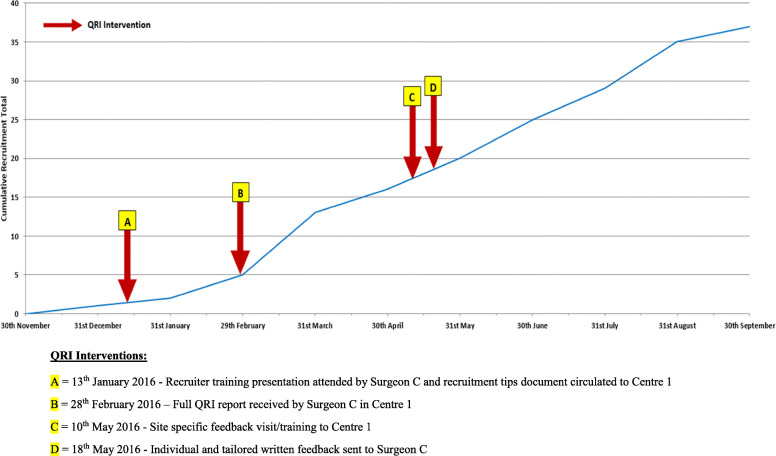
Recruitment to the HAND-1 Study with QRI interventions—centre 1. QRI interventions: (A) 13 January 2016—recruiter training presentation attended by surgeon C and recruitment tips document circulated to centre 1. (B) 28 February 2016—full QRI report received by surgeon C in centre 1. (C) 10 May 2016—site-specific feedback visit/training to centre 1. D 18 May 2016—individual and tailored written feedback sent to surgeon C

**Fig. 3:**
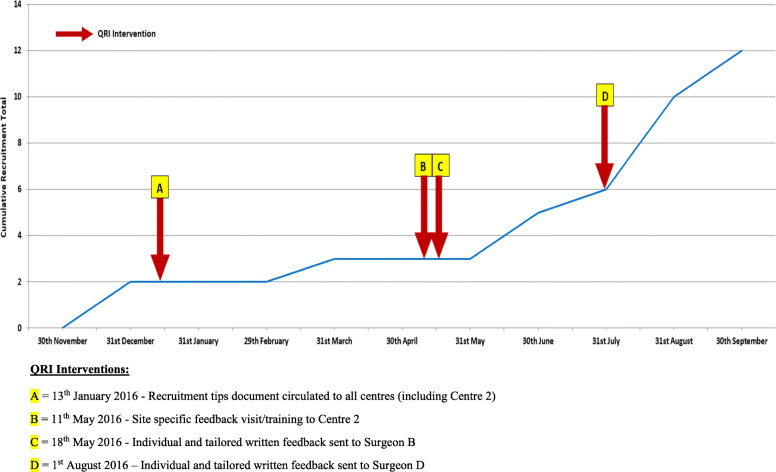
Recruitment to the HAND-1 Study with QRI interventions—centre 2. QRI interventions: (A) 13 January 2016—recruitment tips document circulated to all centres (including centre 2). (B) 11 May 2016—site-specific feedback visit/training to centre 2. (C) 18 May 2016—individual and tailored written feedback sent to surgeon B. (D) 1 august 2016—individual and tailored written feedback sent to surgeon D

HAND-1 successfully reached its recruitment target, with increases in recruitment occurring soon after the delivery of QRI interventions. For example, conversion rates (patients approached/randomised) increased in centre 1 in the months soon after surgeon C had received feedback on recruitment obstacles (Feb 2016, see Table 7 and Fig. 2). Figures from centre 2 suggested increases to conversion rates in the months after their training visit (May 2016, see Table 7 and Fig. 3). This increase appeared to be related to a rise in the number of patients screened, which occurred after suggested changes to the recruitment pathway were implemented. The new recruiting surgeon at centre 2 (surgeon D) also received feedback on his recruitment appointments just prior to this observed increase (August 2016, see Table 7 and Fig. 3). In centre 3, we were unable to provide feedback from consultation recordings, but there was a marked increase in recruitment numbers and conversion rates (Table 7) following the delivery of a tailored recruitment tips document (Table 6) which focused on likely key issues derived from other centres, screening logs and an interview with a centre recruiter (surgeon A). Recruitment figures from all centres, however, showed that increases after interventions appeared to tail off after several months, particularly towards the end of recruitment.

**Table 7:** HAND-1 recruitment figures for all centres

Centre/month	No. screened	No. eligible/approached	No. randomised	Conversion rate (approached/randomised)
**Centre 1**				
Dec 2015/Jan 2016	12	7	**1**	**14%**
Feb–Apr 2016	27	17	**14**	**82%**
May–July 2016	35	23	**13**	**56%**
Aug/Sept 2016	26	16	**8**	**50%**
**Centre 2**				
Dec 2015/Jan 2016	7	4	**2**	**50%**
Feb–Apr 2016	21	11	**1**	**9%**
May–July 2016	65	27	**5**	**18%**
Aug/Sept 2016	26	9	**4**	**44%**
**Centre 3**				
Dec 2015/Jan 2016	8	6	**3**	**50%**
Feb–Apr 2016	20	15	**7**	**46%**
May–July 2016	13	8	**6**	**75%**
Aug–Sept 2016	14	11	**6**	**54%**

Key dates of QRI feedback:

Centre 1:

• 28 February 2016—Full QRI report received by lead recruiter (surgeon C) in centre 1

• 10 May 2016—Site-specific feedback/training visit to centre 1

• 18 May 2016—Individual and tailored written feedback to recruiters in centre 1

Centre 2:

• 11 May 2016—Site-specific feedback training visit to centre 2

• 18 May 2016—Individual and tailored written feedback to lead recruiter (surgeon B) in centre 2

• 01 August 2016—Individual and tailored written feedback to surgeon D in centre 2

A comparison of audio-recordings pre- and post-feedback suggested that recruiters had changed the way they explained the study, in line with guidance from the QRI team. There were instances of surgeon C exploring and balancing patient treatment preferences as advised, specifically using the advantages/disadvantages of a patient’s most/least preferred option to gently challenge their views and emphasise the position of clinical equipoise (Table 8). Similarly, there were examples of surgeon B providing more balanced information on procedures, including comparatively more positive descriptions of LF (Table 8). Such changes though were inconsistent within and across their appointments, but there were signs of behaviour change during the consultation in the limited post-training timeframe.

**Table 8:** Excerpts from recruitment consultations pre and post intervention

**Surgeon C:** **Excerpt from consultation pre-feedback (February 2016)** **Patient 16:** “I think I’d like to have, would prefer to have, surgery.” **Surgeon C:** “No, that’s absolutely fine” **Excerpt from consultation post-feedback (March 2016)** **Patient 22:** “…I would love to do this study but I’m just wondering…what to do because I’m already going to have six weeks and then if I have to have another month off, I might not get paid.” **Surgeon C:** “No, no, that’s fine. That’s okay. So, which one would you?” **Patient 22:** “If I had [needle fasciotomy] it would only be about a week…?” **Surgeon C:** “It would be a shorter period but it’s not so good at getting it straight. Just sometimes they don’t come straight at all and we have to go and do the other one anyway. It’s very difficult and that’s why we’re doing the study because we don’t know which is better” **Patient 22:** “Yes, right. I’ll do the study then… it’s not up to me, it’s down to the study.” **Surgeon B:** **Excerpt from consultation pre-feedback (February 2016)** **Surgeon B:** “Limited fasciectomy has a higher risk of complications but possibly a longer-term disease-free interval; slightly harder to do repeatedly, but you don’t need it doing as often. Needle fasciotomy: faster recovery, smaller risk of complications, but you might need it doing more often” **Excerpt from consultation post-feedback (August 2016)** **Patient 68:** “I’m not sure how long it’ll be for things like driving?” **Surgeon B:** “Needle fasciotomy 5–6 days, but it does come back we know much faster…Limited fasciectomy, we are probably talking about 3–4 weeks to get back to comfortable, safe driving, but we know that it is a much longer time to recurrence”	

## Discussion

This paper has reported on the integration of an intervention (the QRI) to optimise recruitment and informed consent to a feasibility RCT of treatment for Dupuytren’s contracture (the HAND-1 study). Using multiple methods, the QRI identified recruitment obstacles and implemented tailored strategies to address them. There was a steady improvement in recruitment, with some of the more pronounced increases occurring soon after delivery of QRI data feedback and recruiter training. The feasibility study achieved its recruitment target within the required timeframe [17], demonstrating that recruitment to a main trial of NF and LF with integrated QRI is feasible. The QRI study also demonstrated that it is possible for surgeons to be trained to recruit into an RCT of two surgical procedures with very different patient experiences, despite some initial reservations that patient and recruiter equipoise would hinder participation.

Two key recruitment obstacles emerged from the research. Recruitment pathways, in particular how potentially eligible patients were identified, appeared to be restricting the pool of eligible patients, especially in centre 2 where potentially eligible patients were asked to opt-in to a research clinic if they wanted to hear about the study. Recruiter equipoise and accepting of patient preferences at face value also appeared to be impacting recruitment with instances where recruiters were subtly steering patients towards one of the treatments, with or without knowing it, or were not establishing if the patient fully understood the pros and cons of their preferred and non-preferred treatment. Interviews with patients who participated in HAND-1 to explore their experience of treatment and participation [6] triangulates the finding of an imbalanced delivery of information—a few either recalled little information on one of the treatments compared to the other or conveyed that one of the treatment options had been portrayed to them more negatively than the other.

Recommendations from the QRI included changes to the recruitment pathway to ensure that centres were maximising the pool of potentially eligible patients so that as many patients as possible had the opportunity to consider trial participation. Recruiting surgeons were provided with evidence-based training on techniques for balancing treatment discussions and for gently exploring patients’ treatment preferences to ensure patients had full and accurate information [10, 11]. The QRI appeared to lead to positive improvements, as recruitment rates increased in the periods shortly after interventions had been delivered, and recruitment practices seemed to change to promote informed consent. In instances where recruitment numbers did not increase after intervention (for example, interventions C and D at centre 1), recruitment numbers remained stable in the months following the delivery of feedback and training; the interventions may have positively contributed to this achievement. These particular interventions were also preceded by feedback (intervention B) which led to the greatest increase in recruitment numbers at this centre (Fig. 2). Other studies have highlighted similar recruitment obstacles. Pathway issues were identified in a surgical trial for bladder cancer, resulting in simplification of the pathway to reduce the number of professionals that patients encountered prior to being invited to join the RCT [22]. Patient treatment preferences were a commonly cited obstacle by recruitment staff in other studies [14, 23]. These studies noted that recruiters believed patient treatment preferences were responsible for poor recruitment, and they often did not realise their own influence on patients’ treatment decisions during discussions and the impact of this on recruitment. Rooshenas et al. [10] found that equipoise was omitted or undermined in almost half of recruitment appointments (48/105), even though most recruiters considered themselves to be in equipoise. This stemmed from an imbalanced or biassed presentation of the treatment options. These studies, and ours, emphasise the importance of targeted training to raise awareness and help clinicians to have more full and open conversations with patients on RCT participation. Similar targeted training/guidance in other studies has led to increases in recruitment and informed consent [11].

This is the first study to explore and address recruitment barriers in-depth in a feasibility study of treatment for Dupuytren’s contracture. The strength of the QRI was the identification of common obstacles across centres and obstacles that were unique to individual recruiters/centres, all of which appeared to affect recruitment. This allowed focused feedback on the most salient issues for each centre and individual. All data collection and analysis occurred as recruitment was underway, enabling strategies to be devised and implemented to impact subsequent recruitment.

A limitation of this study is that analysis focuses on two recruiters in two centres. However, the limited data gathered from centre 3 largely from screening logs suggested that the recruitment obstacles there were like those in centres 1 and 2, and those observed in other QRI studies [14]. As with any before-after observational research, it is difficult to determine the cause and effect of the QRI on improvements to recruitment practices. Other, confounding, factors may have been responsible for the observations, such as increased recruiter concentration during appointments due to an awareness of being audio-recorded. However, interventions often immediately preceded increases in recruitment numbers, and an analysis of before-after QRI phase 2 actions within five RCTs (including HAND-1) showed promising evidence to suggest that the actions designed to address issues around approaching patients in the HAND-1 RCT (centre 2) led to significant improvements in the number of patients approached per centre per month [16]. Our analysis has important implications for a full RCT comparing outcomes after surgical interventions, as the recruitment obstacles identified here can be minimised in the main trial, for example, by advising on efficient methods of screening for potentially eligible patients. It has highlighted the fragility of recruitment processes [24] and the need for continued training for recruiters throughout RCTs [25], as otherwise the effects of training/guidance can tail off over time and emerging new challenges can go undetected. This emphasises the importance of the continued integration of the QRI in the full RCT. The study has highlighted lessons that might be useful to other surgical RCTs, such as the value of enabling recruitment processes to maximise the identification of potentially eligible participants and ensuring that recruiters convey equipoise in their description of study procedures and explore patient treatment preferences to confirm fully informed decision-making.

## Conclusions

The integration of the QuinteT Recruitment Intervention into a feasibility study of surgical treatments for Dupuytren’s contracture (the HAND-1 study) identified obstacles relating to recruitment and informed consent that were amenable to change through raising awareness, sharing of good practice and training throughout the recruitment period. The HAND-1 study was subsequently successful in reaching its recruitment target within the required timeframe. The study has important implications for the main trial and other surgical or similar trials that are anticipated to encounter issues around patient and recruiter equipoise of treatments and variation in recruitment pathways across centres. Our study has also emphasised the benefits of including a feasibility study in a trial’s design, with a clear objective to explore and respond to issues which may impact negatively on recruitment in preparation for the main trial [4, 26]. Embedding a QRI in an RCT, at feasibility or main stage, offers an opportunity for a detailed and nuanced understanding of key recruitment challenges and the chance to address them in “real-time” as recruitment proceeds.

## Supplementary Information

**Additional file 1.** Hand-1 Quintet Recruitment Intervention (QRI) Staff Interview Topic Guide.

## Data Availability

Data sharing is not applicable to this article as no datasets were generated or analysed during the current study.

## Abbreviations

LF: Limited fasciectomy
NHS: National Health Service
NF: Needle fasciotomy
NCTU: Nottingham Clinical Trials Unit
PIS: Patient information sheet
QRI: QuinteT Recruitment Intervention
TMG: Trial management group
RCT: Randomised controlled trial
