# Supplementary material for: Optimising recruitment to the HAND-1 RCT feasibility study: integration of the QuinteT Recruitment Intervention (QRI)
Source: Pilot Feasibility Stud. 2020 Nov 9;6:173. doi: 10.1186/s40814-020-00710-1 (PMC7650179; doi:10.1186/s40814-020-00710-1)
Supplement: Supplementary file 1 — Additional file 1. Hand-1 Quintet Recruitment Intervention (QRI) Staff Interview Topic Guide. [file 40814_2020_710_MOESM1_ESM.doc]

**Hand-1 Quintet Recruitment Intervention (QRI)**

**Staff Interview Topic Guide**

1. **Background**

- Can you describe your current position and role in the study?

- Do you have prior experience of working in RCTs?

- Could you explain what the HAND-1 study is about?

- Do you think there is a need for this trial or not?

- Could you tell me how and why you got involved in HAND-1?

- Are you aware of any prior evidence relating to this area of research?

1. **HAND-1 recruitment pathway in site**

- Can you talk me through what happens from the time a patient is referred to your centre to the time a decision is made about whether or not they participate in the trial?

1. **Eligibility criteria**

- Could you describe the eligibility criteria to me? *Probe for thoughts on the criteria teasing out any areas of concern they may have*

1. **Introducing/explaining the trial**

- How do you introduce/explain the HAND-1 study to patients? *Probe around randomisation, uncertainty and description of treatments, and patient responses/understanding*

1. **Reasons for declining participation**-Why do patients decline to participate in this trial? *Probe reasons behind decline and how recruiters respond to this*
2. **Main recruitment barriers in HAND-1**
   - How do you think recruitment is going so far?
   - What would you say are the main difficulties you face as a recruiter?
   - Can you describe specific examples of ‘good’ and ‘less good’ recruitment experiences?- Do you feel recruitment is well organised or could be better?

- Do you feel there are difficulties with any particular arm?

1. **Improving recruitment**
   - What might, in your opinion, improve recruitment?

**Reiterate thanks and confidentiality of material**
